# Ashkenazi Jewish Centenarians Do Not Demonstrate Enrichment in Mitochondrial Haplogroup J

**DOI:** 10.1371/journal.pone.0003425

**Published:** 2008-10-16

**Authors:** Liran I. Shlush, Gil Atzmon, Roni Weisshof, Doron Behar, Guenady Yudkovsky, Nir Barzilai, Karl Skorecki

**Affiliations:** 1 Ruth & Bruce Rappaport Faculty of Medicine and Research Institute, Technion – Israel Institute of Technology, Haifa, Israel; 2 Molecular Medicine Laboratory, Rambam Health Care Campus, Haifa, Israel; 3 Institute for Aging Research, Diabetes Research and Training Center, Department of Medicine, Albert Einstein College of Medicine, Bronx, New York, United States of America; Tel Aviv University, Israel

## Abstract

**Background:**

Association of mitochondrial haplogroup J with longevity has been reported in several population subgroups. While studies from northern Italy and Finland, have described a higher frequency of haplogroup J among centenarians in comparison to non-centenarian, several other studies could not replicate these results and suggested various explanations for the discrepancy.

**Methodology/Principal Findings:**

We have evaluated haplogroup frequencies among Ashkenazi Jewish centenarians using two different sets of matched controls. No difference was observed in the haplogroup J frequencies between the centenarians or either matched control group, despite adequate statistical power to detect such a difference. Furthermore, the lack of association was robust to population substructure in the Ashkenazi Jewish population. Given this discrepancy with the previous reported associations in the northern Italian and the Finnish populations, we conducted re-analysis of these previously published data, which supported one of several possible explanations: i) inadequate matching of cases and controls; ii) inadequate adjustment for multiple comparison testing; iii) cryptic population stratification.

**Conclusions/Significance:**

There does not exist a universal association of mitochondrial haplogroup J with longevity across all population groups. Reported associations in specialized populations may reflect genetic or other interactions specific to those populations or else cryptic confounding influences, such as inadequate matching attributable to population substructure, which are of general relevance to all studies of the possible association of mitochondrial DNA haplogroups with common complex phenotypes.

## Introduction

Studies of human mtDNA sequence variation have revealed a remarkable partitioning of mtDNA haplogroups with geographic origin of populations. Among Europeans, 95% of the population belongs to 1 of 10 major haplogroups: H, I, J, K, M, T, U, V, W, and X[1]. While the phylogeographic correlation of the mtDNA haplogroups and geographic origins is very well established, the question of whether the distribution of human mtDNA haplogroups is the result of adaptation to the environment or the result of random genetic drift with some modification by purifying selection is still under debate[2]. While most of the homoplasmic substitutions (variations in mtDNA sequence affecting all mtDNA molecules within a given individual) at deeper branches of the mtDNA tree (among them, the haplogroup defining substitutions), are either synonymous mutations, or appear in non-coding regions of the mtDNA[2]; and are most probably selectively neutral, others such as the Asian F haplogroup defining mutation 12406 alter a moderately conserved amino acid[3]. Most of the studies describing an association between mtDNA sequence variants and a well-defined phenotype have focused on pedigree analysis, and have reported heteroplasmic mutations (mutations that are present on some, but not all, copies of the mtDNA sequence within a given individual)[4], or rare homoplasmic variants. Consideration of the possible phenotypic effects of non-synonymous homoplasmic substitutions that define deeper mtDNA phylogeographic branch-points has suggested that such substitutions have either not yet been eliminated by purifying selection, or are not deleterious[2], and might even be beneficial. A number of association studies reported positive associations between mtDNA homoplasmic substitutions and various phenotypes[5]–[8]. One specific association that has been reported is that of longevity with haplogroup J in both a northern Italian population[9], a Finnish population[10] and a and a sub-branch of haplogroup J was enriched among Irish centenarians[11]. However other studies in French[12], and southern Italian[13] populations could not replicate these results.

Given the inconsistency among the different studies, we have decided to examine the possible association haplogroup J with longevity in a more genetically refined population of the Ashkenazi Jews (AJ). Several studies have documented the homogeneity of the AJ poulation, attributed to a strong founder effect, followed by a rapid population expansions and a mating pattern characterized by high levels of endogamy[14] and consanguinity[15]. These population attributes enhance the power of genetic association studies for complex phenotypes. Indeed, specific mutations in a number of disease genes (e.g., breast cancer gene, adenomatosis polyposis coli gene, hereditary prostate cancer gene and other) were identified in the AJ population at higher prevalence and replicated in other populations[16], [17]. The propensity of variable genetic background to modify phenotypic expression based on a given genetic variant at a given locus (epistasis) is closely related to the genetic diversity of the study population[18]. Together with locus heterogeneity, low penetrance, variable expressivity, pleiotropy, and limited statistical power[19], such epistatic effects can confound the ability to identify a true association. On the other hand the presence of cryptic population stratification, inadequate matching of cases and controls within the study population, and inadequate adjustment for multiple comparison testing can yield a spurious association[20]. By focusing on a well defined highly inbred population such as AJ, taking into account population substructure, and with adequate matching and statistical analysis adjustments, the genetic determinants of a particular phenotype may be more homogeneous and hence more clearly identified, and conversely false positive associations due to population stratification can be avoided. Therefore, the AJ population seems to provide an excellent opportunity to address the question of a possible association of mitochondrial haplogroups with longevity, and more specifically to understand the possible association between longevity and haplogroup J.

## Results

### Haplogroup J

There was no difference in haplogroup J frequency in the “Longenity Project” control group 10/93 (10.75%), in comparison to the Israeli control group 47/583 (8.06%) p = 0.42. Furthermore haplogroup J frequencies showed no differences between the centenarian, and “Longenity Project” control group, the Israeli control group or the joint control groups (8.3% vs. 10.75%, 8.06%, 8.43%, p = 0.678, 0.68, 0.79, respectively, Table 1).

**Table 1:** Haplogroup frequencies among AJ centenarians and controls.

Haplogroup	Centenarians	%	Spouses AJ	%	Israeli AJ	%
A	1	0.41	0	0.00	1	0.17
H	61	25	22	23.66	119	20.41
HV	19	7.79	8	8.60	34	5.83
R0	5	2.05	3	3.23	16	2.74
I	5	2.05	1	1.08	5	0.86
J	20	8.3	10	10.75	47	8.06
K	70	28.69	27	29.03	186	31.90
L	3	1.23	1	1.08	10	1.72
M	4	1.64	0	0.00	11	1.89
N	16	6.64	4	4.30	59	10.12
T	8	3.28	9	9.68	28	4.80
U	7	2.87	3	3.23	34	5.83
V	13	5.33	5	5.38	19	3.26
W	5	2.05	0	0.00	8	1.37
X	4	1.64	0	0.00	6	1.03
Total	241	100	93	100.00	583	100

### Population substructure

Comparison of the two control groups, yielded no differences in the overall haplogroup frequencies (Table 1) p = 0.254. The comparison of overall mtDNA haplogroup distribution among centenarians compared to each of the control groups (Table 1) did not show any differences.

The overall haplogroup frequencies of Polish AJ and Romanian AJ were similar (p = 0.353) (Table 2), while the overall haplogroup frequencies of Polish AJ were different in comparison to Ru\Uk AJ (p = 0.003), which is statistically significant while considering Bonferroni correction for multiple hypothesis testing (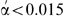). No differences were detected in overall haplogroup frequencies in comparing Romanian AJ and Ru\Uk AJ (p = 0.142). Comparison of the overall mtDNA haplogroup frequency between the “Longenity Project” subject centenarians and Israeli AJ control subgroups (Pol, Rom, Ru\Uk) yielded Pearson chi-square values respectively of p = 0.149; p = 0.695; p = 0.037, and Fisher exact test values respectively of p = 0.535; p = 0.691; p = 0.498.

**Table 2:** Haplogroup frequencies among AJ from various origins.

Haplogroup	Poles AJ	%	Romanian AJ	%	Ru\Uk AJ	%
A	0	0	0	0	0	0
H	22	12.94	24	24.74	33	25.98
HV	13	7.65	6	6.19	11	8.66
R0	2	1.18	1	1.03	7	5.51
I	3	1.76	0	0.00	0	0.00
J	18	10.59	10	10.31	14	11.02
K	65	38.24	29	29.90	21	16.54
L	5	2.94	2	2.06	1	0.79
M	3	1.76	2	2.06	1	0.79
N	10	5.88	11	11.34	11	8.66
T	6	3.53	3	3.09	9	7.09
U	12	7.06	5	5.15	11	8.66
V	6	3.53	1	1.03	5	3.94
W	4	2.35	1	1.03	3	2.36
X	1	0.59	2	2.06	0	0
Total*	170	100	97	100	127	100

Ru = Russians Uk = Ukrainians.

* AJs from other maternal geographic origins have been omitted from this table.

## Discussion

Heritability estimates of longevity derived from twin registries and large population-based samples suggest a significant but modest genetic contribution to human lifespan (heritability ∼15 to 30%)[21]. Associations of mtDNA haplogroup J with longevity have been previously reported for two European populations [9], [10] and one sub-branch of haplogroup J was enriched among Irish centenarians[11]. In contrast studies in other populations could not replicate the association[11]–[13]. We sought to obtain further clarity and insight by examining this association in a population whose genetic structure has been well-characterized and yielded successful associations with genomic loci in a variety of complex phenotypes – namely the AJ population. Furthermore, we took advantage of the availability, extremely well-characterized and adequately powered sample set of centenarians and controls from the “Longenity Project”, and then supplemented this with a further control sample set. Moreover, and of perhaps greatest importance – the subject group has already been used to uncover longevity loci in the genome [22]–[25], giving still further confidence that a negative result for a variant or locus of interest is a true negative. Indeed we were not able to detect an association of longevity with the J haplogroup using this study design.

A number of explanations can be offered to understand the discrepancy. The first and most straightforward is population specificity of the effect. According to this scenario, the association of haplogroup J with longevity might be specific to those populations in which the association was identified, and dependent on epistatic effects with variants elsewhere in the genome in these populations, or with environmental factors applicable to those populations and not to AJ or other populations in which the association could not be replicated. Such an explanation has very important implications, since it would of course motivate the search for such population specific modifiers, and corresponding epistatic or gene-by-environment interactions. However, before launching on such an endeavor it is necessary to consider other possible explanations for the discrepant findings in the different studies.

We have already considered the possibility that the current study was inadequately powered to detect a true association. Power analysis indicated that the likelihood of missing such an association with the current study design was only 20%, for an increase in haplogroup J frequency corresponding to an odds ratio (OR) of 2.

Another possibility is that previously reported associations did not take into account various confounding factors. For example, low counts of haplogroup J in the study of northern Italian centenarians and controls due to small sample size[9], would tend to inflate the Chi^2^ value and could potentially lead to the inference of a false-positive association[26]. The reported association odds ratio (i.e. 15) in this study[9], in the face of estimated 15–30% heritability for this phenotype, suggests that this might have been the case. Thus, even for this very high OR of 15, the power calculated by Fisher's test for the given sample sizes is 74.4%. In the current study we used a much lower OR for the power analysis, and this resulted in the requirement for a much larger sample number for adequate power. Therefore, it is possible that the observed association in the northern Italian study could result from cryptic population stratification, equivalent to inadequate matching of centenarian and control subjects. It has been recently shown that matching for shared geographic origin and ethnic affiliation may not be sufficient[27]–[29], and this is especially important for association with mtDNA, because of the accentuated subregional partitioning of mtDNA haplogroups[30]. For example, recruitment of study participants within a small region or village with relatively low immigration, can easily introduce bias in the mtDNA haplogroup distribution, due to local regional founder effects, i.e., the probability that apparently unrelated members share a common maternal ancestor. In most societies, maternal family name is usually not transmitted to the descendents[30]. Inspecting the populations in the northern Italian study reveals that one of the populations sampled was from an isolated mountainous and large plains region (i.e. Veneto). A phylogenetic study of two villages (Barco and Posina) from this small geographic region revealed notable differences in haplogroup J frequencies (16.7% vs.5.3% respectively)[31]. Furthermore, haplogroup J frequencies in northern Italy shows a remarkable variability: from 3.8% in Gardena[32] 14.5% in Tuscany[1] and 28.5% in Fassa[31]. This large variability in haplogroup J frequency among northern Italians would be expected to accentuate the confounding effects of population stratification on association studies. The overall haplogroup frequency distribution in the northern Italian study was significantly different between male centenarians and male controls, mainly due to an unexpectedly low frequency of haplogroup J and high frequency of haplogroup U among the controls, and it would be of interest to determine if there was a corresponding pattern of geographic subregional difference in the centenarians and controls. Indeed the extreme difference in haplogroup distribution is first and foremost more likely to be explained by differences in regional origin than association with a given phenotype.

A similar concern arises in examining the results of the Finnish longevity study[10]. In comparing the haplogroup J frequency from the joint control groups in that study (33/657), to the haplogroup J frequency in a sample of unrelated Finns 7/49 from a different study[1] a significant difference (p = 0.016), is evident, indicating that population substructure in the Finnish population may have conferred the inference of a causative association of haplogroup J with longevity. This conclusion is further strengthened by comparing the haplogroup distributions within the Finnish population as reported by Torroni et.al[1] to that of the control groups from Neimi et.al[10]. This comparison reveals a statistically significant (p = 0.01) overall haplogroup difference only in comparison with the infant group. In addition, it should be noted that the study of Neimi et al.[10] tested 11 hypotheses, necessitating, correction for multiple hypothesis testing for rejection of the null hypothesis, which in the case of this study would have required p<0.0046[33].

Notwithstanding these concerns – the fact that both the Finnish as well as the northern Italian study found an association of longevity with the same mtDNA haplogroup J certainly motivates further investigation. In this regard, the design of our current study in AJ was powered to identify an OR of 1.75 or higher. Therefore it is certainly possible that a lower genetic contribution of haplogroup J to longevity could not be detected by our current study, and since the exact genetic contribution of mitochondrial variants to the longevity phenotype is unknown and cannot be reliably estimated from previous studies, we certainly cannot exclude this possibility.

While the use of the “Longenity Project” spouse control group dramatically enhances the reliability of matching of cases and controls in terms of population stratification, the same approach potentially introduces a different potential source of bias. As noted above, the AJ population is both consanguineous and endogamous[15]. In an inbred population such as AJ, centenarians and their offspring spouses might share the same mtDNA haplogroup due to consanguinity, a fact that might mask the association between haplogroups and a given trait of interest. The use of the Israeli control group in addition to the spouse based: ”Longenity Project” control group, importantly ameliorates this possible source of bias. The overall absence of differences in mtDNA haplogroup distributions between the “Longenity Project” and Israeli control groups indicates that the matching was successful, regardless of geography and consanguinity.

In a recent study, Feder et.al[29] described geographic substructure among AJ living in Israel. Most of the substructure effect in this study[29] stemmed from differential representation of haplogroups K and H, among AJ originating from Poland, Romania, and former Soviet Union, these results were demonstrated in our AJ samples sets as well (Table 2). Importantly, no difference was reported for haplogroup J frequencies among these three groups in Feder et al.[29], nor in our sample sets. The fact that AJ population substructure did not influence haplogroup J frequency cannot be generalized to other haplogroups or to other genetic loci, and future studies on genetic associations among AJ should take into account the potential influence of cryptic substructure based on European regional origin

While the current study tends to rule out a cross-population or global association of the mtDNA J haplogroup with longevity, it does not rule out other forms of association of mtDNA sequence variation with lifespan. Several studies have tried to relate lower frequency mtDNA polymorphisms to the longevity phenotype[11], [12], [34], [35], or the appearance of somatic mutations in the mitochondria of centenarians[36]. Furthermore, as already noted – we cannot rule out the possibility of population-specific effects of certain mtDNA haplogroups interacting with a given nuclear genomic background or environment. This would require further well-designed studies in other populations, taking into account all of the potentially confounding factors outlined above.

## Materials and Methods

### Study population

This case-control study included 241 centenarians recruited as part of the Albert Einstein College of Medicine “Longenity Project”: http://www.aecom.yu.edu/longenity/page.aspxID2982ekmensel1490_submenu_1492_btnlink, of whom 213 have been previously described[22], [23] with an additional 28 for the current study, and together are termed the “Longenity Project subject group”.

The centenarian ages were defined by birth certificates or dates of birth as documented on passports. The mean age at recruitment was 97.8±2.8, 168 were females and 73 males. Inclusion into the centenarian group required that the subjects be living independently when they were 95 years of age as a reflection of good health, although at the time of recruitment they could be at any level of dependency. The control population consisted of two unrelated sample sets of AJ. The “Longenity Project control group” included 93 individuals (36 males, and 57 females, who were spouses of the offspring of the “Longenity Project subject group”, with 75 of them as described elsewhere [23], and 18 additional recruited for the current study. The mean age at recruitment of this subgroup was 69.4±8.8 years. The second control group consisted of Israeli AJ recruited in Israel as previously described[37], and consists of a total of 583 AJ (417 males, and 166 females) with a mean age at recruitment of 41.8±16.2 year (“Israeli control group”). It should be noted that less than 0.01% of the control group are expected to become centenarians[38], according to previous studies from other populations, and therefore can be considered a suitable control group. All the participants in the current study were interviewed, their maternal origin was reported and they were all self identified as being AJ.

### Genotyping

Sequences of part of the control region were determined from position 16024–300, by the use of the ABI Prism Dye Terminator cycle-sequencing protocols developed by Applied Biosystems (Perkin-Elmer). Haplogroups were defined on the basis of coding region RFLPs and control region polymorphisms. RFLP typing of coding-region sites that are diagnostic for the major mtDNA haplogroups was performed hierarchically beginning with deep-rooting markers of the mtDNA phylogeny[39]. The genotyping of both Israeli and the USA groups has been performed in the Technion laboratory as previously described[37].

### Statistical analysis

Power analysis was conducted using two different methodologies. First, the standard Fisher's test power calculation was conducted using the following parameters: p = 0.05 and an increase in haplogroup J frequency corresponding to an odds ratio (OR) of 2, with the control haplogroup frequency set to 8%[37]. Using these parameters, a sample size of 240 centenarians and 670 controls was needed to yield 80% power. The second power analysis is based on Monte Carlo permutation test that generates power curves for European mtDNA haplogroup studies, which derived a universal equation enabling power calculations for prospective studies. We used this equation in order to calculate power and sample size[40]. The following parameters were used: *N*scaled = 8.5 (corresponding to 90%) N_H_ = 1 (for haplogroup J only), p_0_ = 0.08, and increasing values of p_1_ from 0.09 to 0.16 were used. For p_1_ = 0.13 (corresponding to OR of 1.75) *Ncmin* was 251. For OR greater than 1.75 much higher number of cases and controls were needed.

Equation 1 [40]

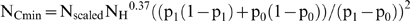

*N*scaled is 8.5 for a p = 0.05, 11 for a p = 0.01, and 14 for a p = 0.001. N_H_ = the number of haplogroups. p_0_ is the frequency of the haplogroup in the control population, and p_1_ is the frequency of the haplogroup in the cases. According to these calculations the number of cases needed is equal to the number of controls[40].

Fisher exact tests were used to verify whether the frequency of haplogroup J was different between centenarians and controls. We used Pearson's chi-square in order to evaluate overall haplogroup frequency differences among the different study populations.

### Population substructure

We utilized two approaches to preempt, assess and take into account possible population stratification among AJ.,. First, as was suggested by Raule *et. al.* [30] population stratification in mtDNA association studies can be avoided by not concentrating the recruitment within small or isolated areas and by checking that the haplogroup distribution of the control samples matches the expected distribution for that country or region, when available and reliable[30]. Secondly we divided each of our sample sets to the three major maternal geographic origins of AJ as has been previously reported[29] also – Doron's paper). We compared mtDNA haplogroup distribution among these three geographic regions: Poland (Pol), Romania (Rom), and former Soviet Union (Russia (RU) and Ukraine (UK).

### Informed consent

All samples reported were derived from buccal swab or blood cell samples that were collected with written informed consent according to protocols approved by the National Human Subjects Review Committee in Israel, and Institutional Review **b**oard of the Albert Einstein College of Medicine. All informed consent were written.
